# The CAR-HEMATOTOX score as a prognostic model of toxicity and response in patients receiving BCMA-directed CAR-T for relapsed/refractory multiple myeloma

**DOI:** 10.1186/s13045-023-01465-x

**Published:** 2023-07-31

**Authors:** Kai Rejeski, Doris K. Hansen, Radhika Bansal, Pierre Sesques, Sikander Ailawadhi, Jennifer M. Logue, Eva Bräunlein, David M. Cordas dos Santos, Ciara L. Freeman, Melissa Alsina, Sebastian Theurich, Yucai Wang, Angela M. Krackhardt, Frederick L. Locke, Emmanuel Bachy, Michael D. Jain, Yi Lin, Marion Subklewe

**Affiliations:** 1 Department of Medicine III – Hematology/Oncology, LMU University Hospital, LMU Munich, Marchioninistrasse 15, 81377 Munich, Germany; 2 Laboratory for Translational Cancer Immunology, LMU Gene Center, Munich, Germany; 3 German Cancer Consortium (DKTK), Munich Site, and German Cancer Research Center, Heidelberg, Germany; 4 Bavarian Cancer Research Center (BZKF), Munich partner site, Munich, Germany; 5 Department of Blood and Marrow Transplant and Cellular Immunotherapy, Moffitt Cancer Center, Tampa, USA; 6 Division of Hematology, Mayo Clinic, Rochester, MN USA; 7 Hospices Civils de Lyon, Université Claude Bernard Lyon 1, INSERM 1052, Pierre-Bénite, France; 8 Division of Hematology and Oncology, Mayo Clinic, Jacksonville, FL USA; 9 IIIrd Medical Department, Klinikum rechts der Isar and Center for Translational Cancer Research (TranslaTUM), School of Medicine, Technical University of Munich, Munich, Germany; 10 Department of Medicine I, Malteser Hospital St. Franziskus Hospital, Flensburg, Germany

**Keywords:** Chimeric antigen receptor, BCMA CAR-T, Hematological toxicity, Cytopenias, Multiple myeloma, Infections

## Abstract

**Background:**

BCMA-directed CAR T-cell therapy (CAR-T) has altered the treatment landscape of relapsed/refractory (r/r) multiple myeloma, but is hampered by unique side effects that can lengthen hospital stays and increase morbidity. Hematological toxicity (e.g. profound and prolonged cytopenias) represents the most common grade ≥ 3 toxicity and can predispose for severe infectious complications. Here, we examined the utility of the CAR-HEMATOTOX (HT) score to predict toxicity and survival outcomes in patients receiving standard-of-care idecabtagene vicleucel and ciltacabtagene autoleucel.

**Methods:**

Data were retrospectively collected from 113 r/r multiple myeloma patients treated between April 2021 and July 2022 across six international CAR-T centers. The HT score—composed of factors related to hematopoietic reserve and baseline inflammatory state—was determined prior to lymphodepleting chemotherapy.

**Results:**

At lymphodepletion, 63 patients were HT^low^ (score 0–1) and 50 patients were HT^high^ (score ≥ 2). Compared to their HT^low^ counterparts, HT^high^ patients displayed prolonged severe neutropenia (median 9 vs. 3 days, p < 0.001), an increased severe infection rate (40% vs. 5%, p < 0.001), and more severe ICANS (grade ≥ 3: 16% vs. 0%, p < 0.001). One-year non-relapse mortality was higher in the HT^high^ group (13% vs. 2%, p = 0.019) and was predominantly attributable to fatal infections. Response rates according to IMWG criteria were higher in HT^low^ patients (≥ VGPR: 70% vs. 44%, p = 0.01). Conversely, HT^high^ patients exhibited inferior progression-free (median 5 vs. 15 months, p < 0.001) and overall survival (median 10.5 months vs. not reached, p < 0.001).

**Conclusions:**

These data highlight the prognostic utility of the CAR-HEMATOTOX score for both toxicity and treatment response in multiple myeloma patients receiving BCMA-directed CAR-T. The score may guide toxicity management (e.g. anti-infective prophylaxis, early G-CSF, stem cell boost) and help to identify suitable CAR-T candidates.

**Supplementary Information:**

The online version contains supplementary material available at 10.1186/s13045-023-01465-x.

## Background

BCMA-directed CAR-T represents a practice-changing immunotherapy platform for patients with r/r multiple myeloma [1–5]. Still, it is associated with a unique toxicity profile that includes Cytokine Release Syndrome (CRS) and Immune Effector Cell-Associated Neurotoxicity Syndrome (ICANS) [6, 7]. Real-world evidence has further underlined the importance of hematological toxicity, referring to severe and/or prolonged cytopenias, which can persist long after lymphodepleting chemotherapy and resolution of clinical CRS [8–11]. Hematotoxicity not only represents the most frequently encountered grade ≥ 3 toxicity of CAR-T [12], but also substantially contributes to the multimodal immunosuppression (e.g. combined cellular and humoral) that drives infectious complications [13–15]. With advances in toxicity management of CRS and ICANS, fatal infections now represent the most common cause of non-relapse mortality (NRM) following CAR-T therapies [16–18].

Early hematological toxicity occurs as a result of the lymphodepleting chemotherapy applied prior to CAR-T administration and has been reported in other disease settings devoid of CAR T-cells [19]. In addition, the unique feature of CAR-T related hematotoxicity stems from the observation that neutrophil and platelet recovery often follow a biphasic trajectory with transient recovery followed by a second dip [8, 20]. Furthermore, recent reports have linked high-grade CRS and the associated inflammatory markers to prolonged cytopenias, supporting the notion that inflammatory insults play a relevant pathophysiologic role [9, 21]. We previously developed the CAR-HEMATOTOX (HT) score to model CAR-T related hematotoxicity in a r/r large B-cell lymphoma (LBCL) patient cohort [8]. The score is calculated prior to lymphodepletion and integrates factors related to pre-CAR-T hematopoietic reserve (e.g. hemoglobin, absolute neutrophil count [ANC], platelet count) and inflammatory state (e.g. C-reactive protein [CRP], ferritin). Notably, the score was associated with an increased rate of severe infections, particularly bacterial infections, and poor treatment outcomes in LBCL patients receiving commercial CD19-directed CAR-T in the 3rd line setting [17, 22]. However, it remains unclear if the HT score also risk-stratifies for toxicity events and clinical outcomes in r/r multiple myeloma patients receiving idecabtagene vicleucel (ide-cel) or ciltacabtagene autoleucel (cilta-cel). Furthermore, detailed real-world reporting of cytopenia and infection incidence rates following BCMA CAR-T remains scarce.

## Methods

### Patients and data collection

In this multicenter retrospective observational study, we included all patients infused with standard-of-care BCMA-directed CAR-T for r/r multiple myeloma across six international CAR-T centers. Toxicity and survival outcomes were assessed in 113 patients receiving either standard-of-care ide-cel (n = 106) or cilta-cel (n = 7). Patients were treated between April 2021 and July 2022. Lymphodepleting chemotherapy with fludarabine and cyclophosphamide was administered according to the manufacturers’ instructions [1, 2]. Clinical metadata was extracted from medical records and databases with IRB approval (see supplemental methods).

### CAR-HEMATOTOX

The score was calculated prior to lymphodepletion using the online CAR-HEMATOTOX calculator from the German Lymphoma Alliance (GLA): https://www.german-lymphoma-alliance.de/Scores.html. A leniency period of up to three days for laboratory markers was provided [8]. One point was allotted for the following criteria: ANC ≤ 1200/µl, hemoglobin ≤ 9.0 g/dl, platelet count 76–175 G/l, CRP ≥ 3.0 mg/dl, and ferritin 650–2000 ng/ml. Two points were provided for a platelet count ≤ 75 G/l and ferritin ≥ 2000 ng/ml. A sum score of 2 or greater was classified as high risk (HT^high^), a score of 0–1 as low risk (HT^low^).

### Defining hematological toxicity

Severe thrombocytopenia was defined as a platelet count < 50 G/L. Severe anemia was defined as a hemoglobin < 8 g/dL or anemia requiring transfusion with packed red blood cells. Neutropenia was defined on the basis of the joint American Society of Clinical Oncology/Infectious Diseases Society of America (ASCO/IDSA) consensus guidelines for cancer-related infection risk [23]. We assessed the total cumulative duration of severe neutropenia as days with an ANC < 500/µL between days 0–60 [8]. The phenotypes of neutrophil recovery (quick, intermittent, aplastic) were defined as previously described [8].

### Toxicity and infection grading

Grading of CRS and ICANS followed American Society for Transplantation and Cellular Therapy (ASTCT) consensus criteria [24]. Toxicity management followed institutional guidelines [17, 25]. A detailed overview of prophylaxis strategies across the participating centers is outlined in Additional file 1: Table S1. Early infection events (day 0–90) were defined as bacterial, viral or fungal based on microbiologic or histopathologic data, or as a clinical syndrome of infection (e.g. pneumonia, cellulitis, cystitis) based on retrospective chart review. Infection onset was defined as the day of the diagnostic test. The clinical source of infection was allocated based on the combination of clinical symptoms, microbiologic isolates and radiographic findings. In the absence of clinical symptoms and/or microbiologic data, neutropenic fever alone was not considered an infection event. Grading of infection severity was determined on a 5-grade scale as previously described: mild, moderate, severe, life-threatening or fatal [13, 17, 26]. Severe (grade ≥ 3) infections were defined as requiring intravenous anti-infective agents and/or hospitalization.

### Clinical outcomes

Efficacy outcomes were assessed according to the International Myeloma Working Group (IMWG) criteria [27]. Confirmatory testing and imaging to confirm complete response in case of extramedullary disease were not mandated [28]. Kaplan–Meier estimates for progression-free (PFS) and overall survival (OS) were calculated from time of CAR-T infusion. HT score groups (high vs. low) were compared by log-rank test, while a univariate Cox regression was applied to study hazard ratios (HRs) comparing HT risk groups. NRM was defined as death post CAR-T infusion without evidence of relapse or progression.

### Multivariable analyses for the aplastic phenotype, severe infections and survival outcomes

Multivariable analysis was performed as a Cox proportional hazards model for PFS and OS incorporating estimated glomerular filtration rate (eGFR) ≥ 60 ml/min, LDH greater than upper limit of normal, Eastern Cooperative Oncology Group performance status (ECOG PS) of 2 or greater, plasma cell infiltration of the bone marrow greater than 50%, and HT score risk category as input variables. These covariates were also explored in a multivariable binary logistic regression analysis studying either the aplastic phenotype or severe infection as the binary outcome.

### Statistical considerations

Receiver operating characteristic (ROC) analyses were performed to assess test characteristics. Associations between continuous variables were analyzed using the Spearman correlation coefficient (r). Statistical significance between groups was explored by non-parametric Mann–Whitney test for continuous variables and Fisher’s exact test for comparison of percentages. Statistical analysis and data visualization was performed with GraphPad Prism (v9.0), SPSS (IBM, v26.0), or R Statistical Software (v4.1.2).

## Results

### Baseline patient characteristics

Between April 2021 and July 2022, we identified 113 patients treated with ide-cel or cilta-cel. Patient characteristics are provided in Table 1. Median age was 65 years (range 39–81), median ECOG was 1 (95% confidence interval [CI] 0–1), and 30% of patients had high-risk cytogenetic abnormalities (del17p, t(4;14), t(14;16)). The patients had received a median of six prior lines of therapy (95% CI 5–6), including 88% with prior autologous stem cell transplantation (ASCT), reflecting the heavily pretreated nature of this patient cohort. Notably, 42.5% of patients had penta-refractory disease and 37% were exposed to alkylating chemotherapy in the 3 months prior to CAR-T. On the last bone marrow (BM) assessment prior to CAR-T infusion, ≥ 5% and ≥ 50% clonal plasma cells were detected in 46% and 25% of patients, respectively. The majority of patients presented to CAR-T therapy with stable or progressive disease.

**Table 1:** Baseline patient characteristics

Patient characteristics	All patients (n = 113)	HT low (n = 63)	HT high (n = 50)	P
**Demographic features**				
Median age, years (range)	65 (39–81)	65 (46–81)	66 (39–77)	0.36
Sex, female, n (%)	48 (42%)	27 (43%)	21 (42%)	> 0.9
Race, Black vs. not^#^, n (%)	23/102 (23.2%)	10/55 (19.2%)	13/47 (27.7%)	0.34
ECOG PS at lymphodepletion				
Median (95% CI)	1 (0–1)	0 (0–1)	1 (1–1)	** < 0.001**
PS ≥ 2, n (%)	12 (9%)	0 (0%)	12 (24%)	** < 0.001**
Country, USA, n (%)	89 (79%)	47 (75%)	42 (84%)	0.25
**Disease features**				
Extramedullary disease, n (%)	51 (45.1%)	27 (42.9%)	24 (48%)	0.70
Revised ISS stage 3, n (%)	12/79 (15.2%)	3/40 (7.5%)	9/39 (23.1%)	**0.066**
Triple refractory disease, n (%)	92 (81.4%)	53 (84.1%)	39 (78%)	0.47
Penta refractory disease, n (%)	48 (42.5%)	28 (44.4%)	20 (40%)	0.70
Any high-risk cytogenetic abnormality (del(17p), t(4;14), (14;16)), n (%)	31/105 (29.5%)	14/56 (25.0%)	17/49 (34.7%)	0.29
Gain1q, n (%)	28/99 (28.3%)	11/52 (21.2%)	17/47 (36.2%)	0.12
Serum LDH				
Median (U/l), 95% CI	208 (194–218)	197 (188–213)	221 (201–278)	**0.049**
> ULN, n (%)	36 (31.9%)	13 (20.6%)	23 (46%)	**0.005**
Median serum albumin (g/dL), 95% CI	3.8 (3.6–3.9)	3.9 (3.8–4.1)	3.4 (3.2–3.6)	** < 0.001**
Median serum beta-2-microglobulin (mg/L), 95% CI	3.1 (2.6–3.4) n = 83	2.6 (2.3–3.1) n = 45	4.0 (3.1–4.4) n = 38	**0.001**
**CAR product**				
Ide-cel, n (%)	106 (94%)	61 (97%)	45 (90%)	0.24
Cilta-cel, n (%)	7 (6%)	2 (3%)	5 (10%)	
**Prior therapy**				
Median lines of prior therapy, 95% CI	6 (5–6)	6 (5–7)	6 (5–6)	0.19
Prior BCMA-directed therapy, n (%)				
Bispecific or ADC	12/96 (13%)	2/51 (4%)	10/45 (22%)	**0.01**
Exposure to alkylating Chemotherapy in the 3 months before CAR-T, n (%)	42 (37%)	20 (32%)	22 (44%)	0.24
Bridging therapy, n (%)	76 (67%)	40 (64%)	36 (72%)	0.42
Response to bridging therapy, n (%)*				0.66
PR or better	7/67 (10%)	4/33 (12%)	3/34 (9%)	
SD/PD	60/67 (90%)	29/33 (88%)	31/34 (91%)	
Alkylating-based bridging therapy, n (%)	23 (20%)	10 (16%)	13 (26%)	0.24
Prior autologous SCT, n (%)	99 (88%)	52 (83%)	47 (94%)	**0.087**
Prior allogeneic SCT, n (%)	4 (3.5%)	2 (3.2%)	2 (4.0%)	> 0.9
**Kidney function**				
Creatinine clearance—at LD (eGFR)				**0.009**
> 60 ml/min—n (%)	84 (74%)	53 (84%)	31 (62%)	
30–60 ml/min—n (%)	25 (22%)	10 (16%)	15 (30%)	
< 30 ml/min—n (%)	4 (4%)	0 (0%)	4 (8%)	
**BM studies**				
Median plasma cells in BM (%), 95% CI	3 (1.5–11)	2.5 (0.5–5)	15 (1.5–65)	**0.013**
BM plasma Cells > 5%—n (%)	52 (46.0%)	23 (36.5%)	29 (58%)	**0.036**
BM plasma Cells > 50%—n (%)	28 (24.8%)	7 (11.1%)	21 (42%)	** < 0.001**
**CAR-HEMATOTOX Components** [8]				
Median C-reactive protein (mg/dl), 95% CI	0.58 (0.40–0.92)	0.45 (0.37–0.65	1.02 (0.42–2.50)	**0.03**
Median ferritin (ng/ml), 95% CI	211 (120–354)	86 (64–141)	811 (625–1158)	** < 0.001**
Median ANC (G/l), 95% CI	2.47 (1.98–2.84)	2.95 (2.30–3.30)	1.77 (1.31–2.49)	** < 0.001**
Median platelet count (G/l), 95% CI	144 (125–158)	181 (163–209)	63 (49–93)	** < 0.001**
Median hemoglobin (g/dl), 95% CI	10.5 (9.7–11.1)	11.6 (11.1–1.9)	8.5 (8.2–9.2)	** < 0.001**
Median CAR-HEMATOTOX score, 95% CI	1 (1–2)	1 (0–1)	3.5 (3–4)	** < 0.001**

Patient baseline characteristics prior to BCMA-directed CAR T-therapy. If not otherwise stated, the median and 95% confidence interval (95% CI) are provided. P-values < 0.1 are highlighted in bold. If the measurement wasn’t available for all patients, the denominator is indicated in the table. LD: lymphodepletion chemotherapy. ECOG: Eastern Cooperative Oncology Group. R-ISS: Revised International Staging SystemHigh-risk cytogenetics: Includes del(17p), t(4;14) and t(14;16) as per the KarMMa trial and Sonneveld et al., Blood 2016. Triple-refractory disease: Refractory to one IMiD, one PI and daratumumab. Penta-refractory disease: Refractory to lenalidomide, pomalidomide, bortezomib, carfilzomib and daratumumab

^*^Response assessment to bridging therapy according to IMWG response criteria was not available or unknown in 7 HT low and 2 HT high patients

The median CAR-HEMATOTOX score was 1 (95% CI 1–2), including 63 HT^low^ (score 0–1) and 50 HT^high^ (score 2–7) patients. No differences in age, sex, race, country, disease refractoriness, use of bridging therapy, or exposure to alkylating-based bridging therapy were noted between both risk groups (Table 1). However, we found that HT^high^ patients were more likely to have an ECOG PS ≥ 2 (24% vs 0%, p < 0.001) and higher Revised International Staging System (R-ISS) stage (23.1% vs 7.5%, p = 0.066). Compared to the HT^low^ group, HT^high^ patients were more likely to have received prior BCMA-directed therapy (22% vs 4%, p = 0.01) and experienced impaired renal function manifesting as reduced creatinine clearance (CrCl) at lymphodepletion (CrCl < 60 mL/min: 38% vs. 16%). Relative to HT^low^ patients, HT^high^ patients more frequently exhibited BM infiltration (≥ 50% plasma cells: 42% vs. 11%, p < 0.001; Table 1) and more frequently received a prior ASCT (94% vs. 83%, p = 0.087), providing a correlate for the more extensive baseline cytopenia. For example, the median hemoglobin was 8.5 g/dL (95% CI 8.2–9.2 g/dL), median platelet count was 63 G/L (95% CI 49–93 G/L), and median ANC was 1.77 G/L (95% CI 1.31–2.49 G/L) for the HT^high^ group. As expected, HT^high^ patients exhibited elevated systemic inflammatory markers, including a median serum CRP of 1.02 mg/dL (95% CI 0.42–2.50 mg/dL) and median serum ferritin of 811 ng/mL (95% CI 625–1158 ng/mL) at time of lymphodepletion.

### Influence of the CAR-HEMATOTOX score on hematological toxicity

**Fig. 1:**
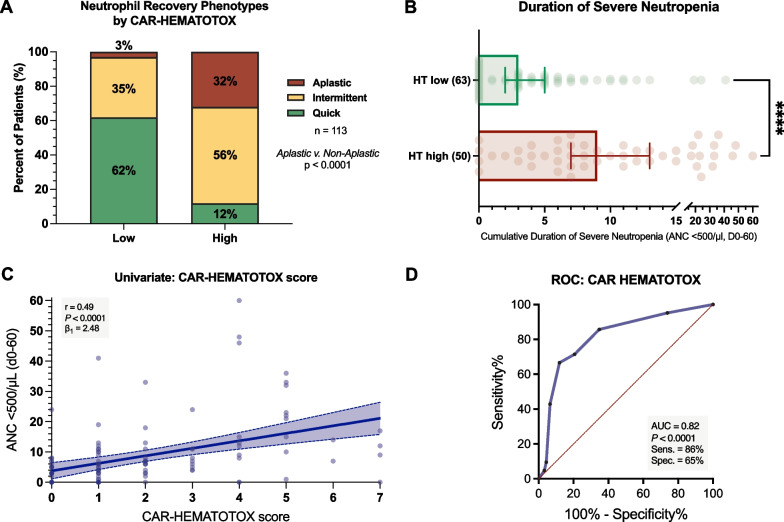
The CAR-HEMATOTOX score identifies patients at risk for severe hematotoxicity. **A** Relative distribution of neutrophil recovery phenotypes by CAR-HEMATOTOX score. Quick: sustained neutrophil recovery without a second dip below an ANC < 1000/µL. Intermittent: neutrophil recovery (ANC > 1500/µl) followed by a second dip with an ANC < 1000/µL after day 21. Aplastic: continuous severe neutropenia (ANC < 500/µL) ≥ 14 days. **B** Median duration of severe neutropenia (ANC < 500/µL) between days 0 and + 60 by CAR-HEMATOTOX score with whiskers indicating the 95% CIs. P-value determined by Mann–Whitney U test (****p < 0.0001). **C** Univariate analysis comparing the CAR-HEMATOTOX score with the duration of severe neutropenia (ANC < 500/µL) between CAR infusion and day + 60. The Spearman correlation coefficient and respective p-value is provided. The calculated slope (β_1_) of the linear regression curve is shown, indicating an average increase in the duration of severe neutropenia of 2.48 days for every increase of 1 in the score. Light shading indicates the 95% confidence bands of the best-fit lines from the simple linear regression. **D** Receiver operating characteristic (ROC) analysis studying the influence of the HT score on the binary outcome of severe neutropenia ≥ 14 days vs. 0–13 days. The area under the curve (AUC), p-value, and test characteristics (sensitivity, specificity) are provided

The proportion of patients displaying aplastic neutrophil recovery was markedly increased in the HT^high^ group (32% vs. 3%, p < 0.0001; Fig. 1A). Importantly, the HT score remained an independent risk factor for the aplastic phenotype when adjusting for other baseline risk factors (adjusted OR [aOR] 10.8, 95% CI 1.9–60.4, p = 0.003; Additional file 1: Fig. S1A). Of interest, ≥ 50% plasma cell infiltration of the bone marrow also independently increased the probability of aplastic neutrophil recovery (aOR = 6.6, 95% CI 1.9–22.9, p = 0.007). The median duration of severe neutropenia (ANC < 500/µL) was significantly longer in HT^high^ patients compared to their HT^low^ counterparts (9 vs. 3 days, p < 0.0001; Fig. 1B). We observed a significant positive correlation between the HT score and the duration of severe neutropenia on univariate analysis (r =  + 0.49, p < 0.0001, β_1_ = 2.48; Fig. 1C). On ROC analysis, we confirmed the discriminatory capacity of the HT score in regard to the previously validated endpoint of severe neutropenia ≥ 14 days (AUC = 0.82, p < 0.0001, sensitivity = 86%, specificity = 65%; Fig. 1D).

HT^high^ patients experienced significantly higher rates of severe thrombocytopenia (78% vs 27%, p < 0.0001 and 56% vs. 6.3%, p < 0.0001), anemia (84% vs. 22.2%, p < 0.0001 and 48% vs. 9.5%, p < 0.0001), and neutropenia (84% vs. 63.5%, p = 0.02 and 36% vs. 6.3%, p < 0.0001) compared to HT^low^ patients within 30 and 100 days post BCMA-directed CAR T-cell therapy, respectively (Table 2). The rates of severe protracted (ANC < 500/µL for ≥ 7 days: 46% vs. 7.9%, p < 0.0001), profound (ANC < 100/µL: 42% vs 19%, p = 0.01), profound protracted (ANC < 100/µL for ≥ 7 days: 14% vs 0%, p = 0.003) and prolonged (ANC < 1000/µL after day + 21: 72% vs 33.3%, p < 0.0001) neutropenia were significantly higher in HT^high^ vs HT^low^ patients, respectively. HT^high^ patients more frequently required transfusions for both platelets (58% vs. 6.3%, p < 0.0001 and 22% vs. 3.2%, p = 0.002) and packed red blood cells (pRBC: 78% vs. 20.6%, p < 0.0001 and 38% vs. 6.3%, p < 0.0001) within 30 and 100 days post BCMA-directed CAR-T, respectively. A trend was observed for increased granulocyte colony stimulating factor (G-CSF) use in HT^high^ versus HT^low^ patients (62% vs. 44.4%, p = 0.087). Thrombopoietin (TPO) agonists were more commonly used in the HT^high^ cohort (14% vs. 4.8%, p = 0.01) and a similar trend was observed regarding the increased use of CD34 + stem cell boosts (6% vs. 2.7%, p = 0.084).

**Table 2:** Hematotoxicity and management

Characteristic	All Patients (n = 113)	CAR-HEMATOTOX Score		p
		Low (n = 63)	High (n = 50)	
**Severe thrombocytopenia (Platelet Count < 50 G/L)**				
Day 0–30	56 (49.6%)	17 (27.0%)	39 (78%)	** < 0.0001**
Day 31–100	32 (28.3%)	4 (6.3%)	28 (56%)	** < 0.0001**
**Severe anemia (Hb < 8 g/dL or requiring transfusion)**				
Day 0–30	56 (49.6%)	14 (22.2%)	42 (84%)	** < 0.0001**
Day 31–100	30 (26.5%)	6 (9.5%)	24 (48%)	** < 0.0001**
**Neutropenia**				
*Phenotype of neutrophil recovery*				** < 0.0001**
Quick	45 (39.8%)	39 (61.9%)	6 (12%)	
Intermittent	50 (40.2%)	22 (34.9%)	28 (56%)	
Aplastic	18 (15.9%)	2 (3.2%)	16 (32%)	
*Severe (ANC* < *500/µL)*				
Day 0–30	82 (72.6%)	40 (63.5%)	42 (84%)	**0.02**
Day 31–100	22 (19.5%)	4 (6.3%)	18 (36%)	** < 0.0001**
*Protracted, severe* (ANC < 500/µL for ≥ 7 days)	28 (24.8%)	5 (7.9%)	23 (46%)	** < 0.0001**
*Profound (ANC* < *100/µL)* Day 0–100	33 (29.2%)	12 (19.0%)	21 (42%)	**0.01**
*Protracted, profound* (ANC < 100/µL for ≥ 7 days)	7 (6.2%)	0 (0%)	7 (14%)	**0.003**
*Prolonged* (ANC < 1000/µL measured ≥ 21 days after CAR-T)	57 (50.4%)	21 (33.3%)	36 (72%)	** < 0.0001**
**Supportive therapies—n (%)**				
*Platelet transfusion*				
D1–30	33 (29.2%)	4 (6.3%)	29 (58%)	** < 0.0001**
D30–100	13 (11.5%)	2 (3.2%)	11 (22%)	**0.002**
*pRBC transfusion*				
D1–30	52 (46.0%)	13 (20.6%)	39 (78%)	** < 0.0001**
D30–100	23 (20.4%)	4 (6.3%)	19 (38%)	** < 0.0001**
*Granulocyte colony stimulating factor (G-CSF) use*	59 (52.2%)	28 (44.4%)	31 (62%)	**0.087**
First day of G-CSF—median (range)	9 (1–114)	9 (3–30)	9 (1–114)	0.96
Last day of G-CSF—median (range)	22.5 (7–149)	21 (7–149)	27 (7–137)	0.29
*Thrombopoetin (TPO) agonist use*	10 (8.8%)	3 (4.8%)	7 (14%)	**0.01**
First day of TPO agonist—median (range)	36 (4–125)	41 (37–42)	33 (4–125)	0.38
Last day of TPO agonist—median (range)	42 (6–223)	101 (37–140)	42 (33–223)	0.50
*CD34 + Stem cell boost*	3 (2.7%)	N/A	3 (6%)	**0.084**
Day of boost (range)	84 (71–118)	N/A	84 (71–118)	
Dose of boost (CD34 + cells × 10^6^/kg)—median (range)	2.6 (2.41–2.97)	N/A	2.6 (2.41–2.97)	
*IVIG use*	24 (21.2%)	16 (25.4%)	8 (16%)	> 0.9

Distribution of metrics of hematological toxicity and concomitant management in the first 100 days after ide-cel or cilta-cel infusion comparing CAR-HEMATOTOX (HT) high vs. low patients. P-values determined by Mann–Whitney test for continuous variables and Fisher’s exact tests for categorical variables; p-values < 0.1 are highlighted in bold

### The CAR-HEMATOTOX score identifies patients at risk for ICANS, early infections and non-relapse mortality

While severe CRS rates (grade 3 or higher) were numerically higher in HT^high^ versus HT^low^ patients (10% vs 2%, p = 0.14), no statistically significant difference was observed. Both the rate of mild-to-moderate ICANS (18% vs. 9%) and especially severe ICANS (16% vs. 0%, p < 0.001) was higher in HT^high^ patients, likely resulting in the increased utilization of glucocorticoids (52% vs. 29%, p = 0.01; Additional file 1: Table S2). On the other hand, the anti-IL-6 receptor antagonist tocilizumab and anti-IL-1 receptor antagonist anakinra were employed at a similar rate across both risk groups. A trend towards more frequent intensive care unit (ICU) admissions was noted in HT^high^ compared to HT^low^ patients (10% vs 1.6%, p = 0.086). Overall, the increased toxicity burden observed in the HT^high^ cohort translated into a longer median duration of hospitalization (13 vs. 8 days, p < 0.0001; Additional file 1: Fig. S2).

**Fig. 2:**
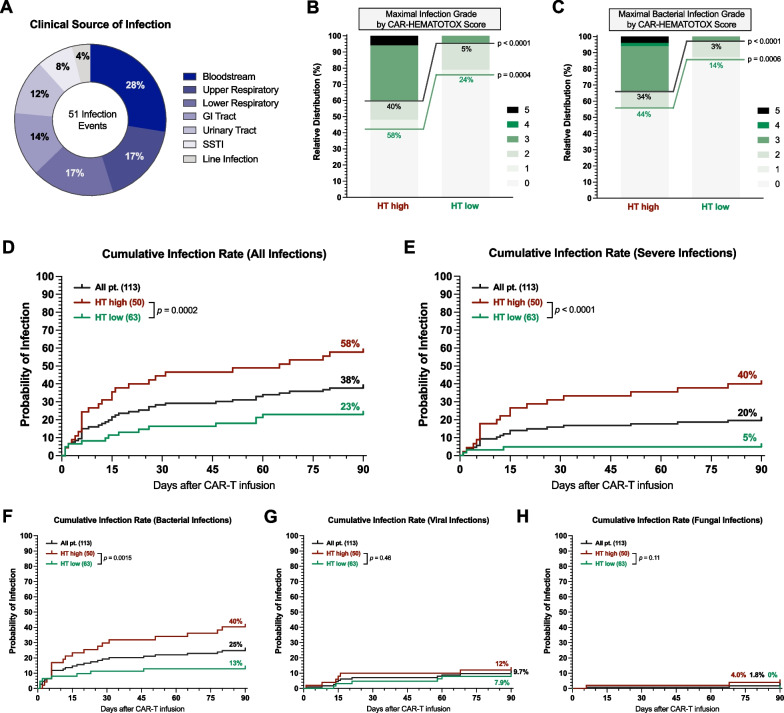
The CAR-HEMATOTOX score identifies patients at risk for severe infectious complications. **A** Clinical source of infection of the 51 infection events. **B**–**C** Relative distribution of infection grades for all infection subtypes (**B**) and bacterial infections only (**C**) comparing HT high versus low patients. Infection grades (1°–5°) are color-coded in shades of green with the connecting green and gray lines and percentage numbers comparing all-grade and grade ≥ 3 infections, respectively, in HT high versus low patients. Significance values were determined by Fisher’s exact test (*p < 0.05, **p < 0.01, ***p < 0.001, ****p < 0.0001). **D**–**H** Cumulative incidence curves (D0–90) by HT score for any-grade (**D**), grade ≥ 3 (**E**), as well as bacterial (**F**), viral (**G**), and fungal (**H**) infections. Comparison of HT risk groups was performed by log-rank test

During the first 90 days following BCMA-directed CAR T-cell therapy, we observed a total of 51 infection events in 44 patients (39%). Bloodstream infections represented the most common infection source (28%), followed by upper/lower respiratory (17%, respectively) and gastrointestinal tract infections (14%) (Fig. 2A). Infections of any-grade were more common in the HT^high^ cohort (58% vs. 23%, p = 0.0002; Fig. 2B). This was particularly evident for severe infections (40% vs. 5%, p < 0.0001), including 1 fatal fungal infection (death on day + 65) and 2 fatal bacterial infections (days + 6 and + 26) in HT^high^ patients. Severe bacterial infections were markedly more frequent in the HT^high^ cohort (34% vs. 3%, p < 0.0001; Fig. 2C). Conversely, no life-threatening (grade IV) or fatal (grade V) infections were noted in the HT^low^ group. Concomitantly, the cumulative 90-day rate of any-grade and severe infections was increased in the HT^high^ patients (Fig. 2D, E). The cumulative rate of bacterial infections was also higher in the HT^high^ compared to the HT^low^ cohort (40% vs. 13%, p = 0.0015). However, no significant differences were observed between groups in the cumulative viral (12% vs. 7.9%, p = 0.46) and fungal (4.0% vs 0%, p = 0.11) infection rates (Fig. 2F–H). Notably, multivariable analysis identified the HT score to be an independent predictor for the development of severe infections (aOR 4.9; 95% CI 1.1–21.4; p = 0.03; Additional file 1: Fig. S1B).

Seven (6.2%) patients who received BCMA-directed CAR T-cell therapy died as a result of non-relapse mortality (NRM) by last follow-up: 5 deaths (71.4%) were attributed to infection, 1 (14.3%) was attributed to grade 5 CRS, and 1 (14.3%) was a result of cardiotoxicity (cardiomyopathy) (Additional file 1: Fig. S3A). On the other hand, 20 patients died of multiple myeloma progression during the first year after CAR-T infusion. One-year NRM for the entire cohort was 6.9% and was significantly increased in HT^high^ versus HT^low^ patients (12.7% vs. 2.1%, p = 0.019; Additional file 1: Fig. S3B).

### Prognostic influence of the CAR-HEMATOTOX score on response to therapy and survival

**Fig. 3:**
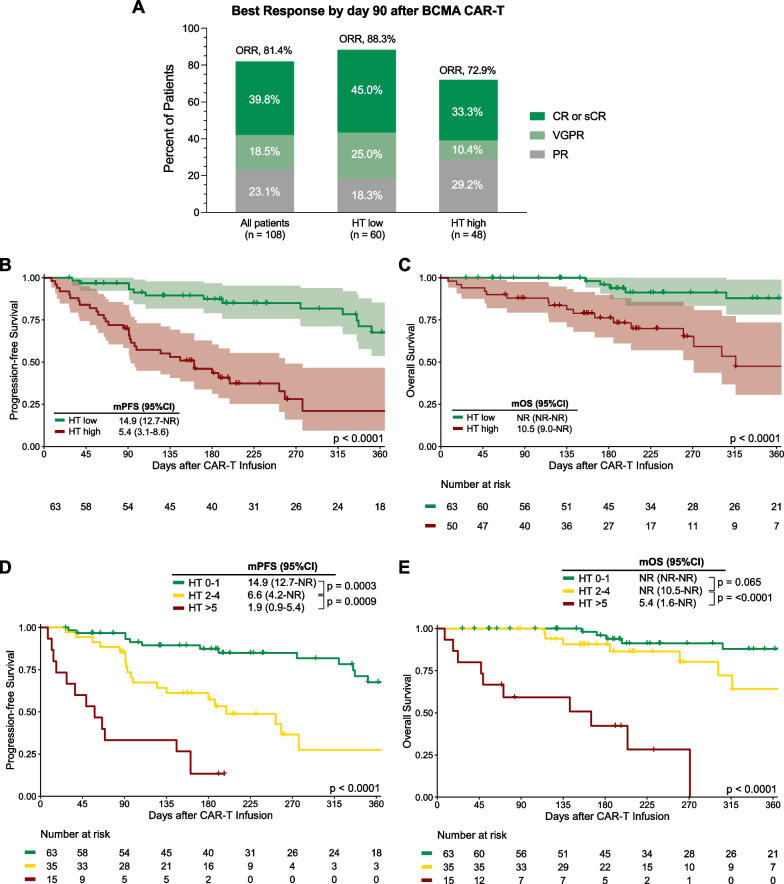
The CAR-HEMATOTOX score identifies patients at risk for poor treatment outcomes. **A** Best overall tumor response at day 90 according to International Myeloma Working Group (IMWG) criteria. **B**–**C** Kaplan–Meier estimates of progression-free survival (PFS, **B**) and overall survival (OS, **C**) comparing HT high versus low patients. **D**–**E** Kaplan–Meier estimates of PFS (**D**) and OS (**E**) comparing low risk (HT score 0–1, green), intermediate to high-risk (score 2–4, yellow), and ultra high-risk patients (score ≥ 5, red). The superimposed table depicts the median and 95% confidence interval of survival estimates, as well as the p-values from the univariate Cox regression. The number at risk at each follow-up time point ist depicted below the x-axis. The p-value of the Mantel–Cox log-rank test is provided on the graph inset

Best overall response rate (ORR) by day 90 was assessed in 108 patients as 5 patients who were in active follow-up had not reached this time point and/or did not have an evaluable response assessment. A high HT score was associated with inferior ORR (72.9% vs. 88.3%, p = 0.048) and inferior very good partial response (VGPR) rates (43.8% vs. 70.0%, p = 0.01), but not complete response (CR) or stringent CR rates (33.3% vs. 45.0%, p = 0.24) (Fig. 3A; Additional file 1: Table S3). After a median follow-up of 7.9 months, the median PFS for the entire cohort was 11.2 months (95% CI 8.6 months—not reached) and the median OS was not reached (Additional file 1: Fig. S4). In evaluable patients, 1-year PFS was 47% (95% CI 36–61%) and 1-year OS was 71% (95% CI 61–83%).

**Fig. 4:**
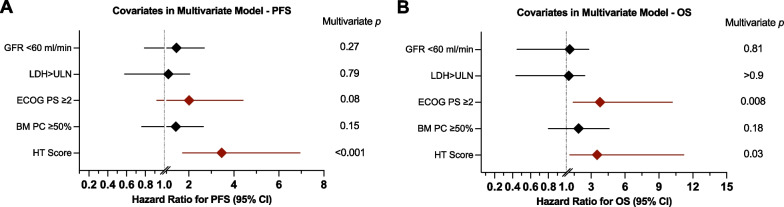
The CAR-HEMATOTOX score represents an independent adverse risk marker for PFS and OS on multivariable analysis. Forest plots of the multivariable Cox regression analysis for PFS (**A**) and OS (**B**) adjusted for the baseline risk factors of poor renal function (eGFR < 60 ml/min), LDH greater than upper limit of normal, ECOG performance status 2–4, plasma cell infiltration of the bone marrow greater than 50%, as well as HT risk group (high vs. low). Adjusted p-values accounting for the respective covariates are displayed on the graph inset. Variables reaching a p-value < 0.1 are highlighted in red (increased hazard ratio)

Compared to their HT^low^ counterparts, HT^high^ patients displayed inferior PFS (median PFS 5.4 vs. 14.9 months, respectively; p < 0.0001; Fig. 3B) and OS (median OS 10.5 months vs. not reached, respectively; p < 0.0001; Fig. 3C). In the HT^high^ cohort, those with a HT score ≥ 5 had particularly poor PFS (median 1.9 vs. 6.6 months respectively; p = 0.0009; Fig. 3D) and OS (5.4 vs not reached, respectively; p < 0.0001; Fig. 3E) relative to the patients with a HT score of 2–4. Multivariable Cox proportional hazards modelling adjusting for other established adverse risk factors showed that a high HT score represented an independent adverse risk marker of both PFS (aHR 3.5, 95% CI 1.7–6.9, p < 0.001; Fig. 4A; Additional file 1: Table S4) and OS (aHR 3.5. 95% CI 1.1–11.2, p = 0.03; Fig. 4B; Additional file 1: Table S5). Furthermore, poor ECOG PS represented an independent poor risk factor of OS (p = 0.008), with a trend observed for PFS (p = 0.08).

## Discussion

In this multi-center international study, we describe a high real-world incidence of both hematological toxicity and early infections in 113 patients receiving BCMA CAR-T for r/r multiple myeloma. Furthermore, we demonstrate that the CAR-HEMATOTOX score identified high-risk candidates for severe toxicity events prior to lymphodepleting chemotherapy. Of interest, high HT scores were also associated with inferior response rates at day 90 and poor survival outcomes.

Overall, the incidence of hematological toxicity in our multiple myeloma cohort was slightly lower compared to a pooled analysis of 235 r/r LBCL patients employing a similar methodology [8]. Interestingly, severe CRS was less frequent in the myeloma compared to the LBCL cohort, and the myeloma patients also exhibited lower levels of systemic inflammation prior to lymphodepletion [8, 9, 21, 29]. One likely hypothesized reason for the lower rate of hematotoxicity may lie in the antigen target (BCMA vs. CD19), as BCMA is preferentially expressed by mature B-lymphocytes with minimal expression in hematopoietic stem cells [30–32], while CD19 is commonly expressed on marrow-derived B-cell progenitors [33]. This may result in less extensive on-target/off-tumor toxicity within the bone marrow niche for BCMA-directed CAR T-cells. Nonetheless, the cytopenia rate was still substantial in our cohort, with cytopenia representing the most frequent CTCAE grade ≥ 3 toxicity, which we observed in approximately 75% of our myeloma patients during the first 100 days following CAR-T infusion. For this reason, future clinical trials should include detailed reporting on both the quantity (e.g. depth, duration) and quality (e.g. biphasic vs. monophasic, phenotypes) of post-CAR-T cytopenias. To this end, a harmonized consensus grading system has been developed for immune effector cell-associated hematotoxicity (ICAHT) by the European Hematology Association (EHA) and European Society for Blood and Marrow Transplantation (EBMT) [34, 35].

Importantly, the degree of cellular immunosuppression conferred by profound and prolonged cytopenia likely plays a critical role in predisposing myeloma patients for infectious complications. Indeed, the large majority of infections, particularly severe and bacterial infections, were observed during the first 30 days during the phase of coincident cytopenia and CRS/ICANS, consistent with prior reports [10, 36]. The high incidence of bacterial infections in the HT^high^ patients highlights the link between the duration and depth of neutropenia and subsequent development of infections. While the incidence of viral infections during the first 90 days was low in our cohort at 10%, these infections are typically observed at a later time point as a consequence of prolonged B-cell aplasia and consecutive hypogammaglobulinemia [14]. Notably, infections were the main determinant of non-relapse mortality. With advances in CRS management and the associated decrease of severe CRS, myeloma patients are thus more likely to die of infectious causes than CRS or ICANS following CAR-T therapy. To mitigate the clinically relevant risk of infections, physicians may consider the use of early and/or prophylactic G-CSF. For example, Lievin and colleages recently reported that early G-CSF prophylaxis on day + 2 was safe, did not impact CAR-T expansion kinetics, and reduced the rate of febrile neutropenia [37]. In a further retrospective report by Miller et al., early G-CSF resulted in faster neutrophil recovery, and was not associated with a significant differences in toxicity in myeloma patients [38]. Furthermore, broad anti-infective prophylaxis (including the use of fluoroquinolones and mold-active azoles) during the early phase of CAR-T therapy may reduce the rate of severe infections, though prospective studies are needed to shed light on potential harmful sequelae. Finally, stem cell boosts, generated from either an autologous or allogeneic source, represent a safe and clinically feasible strategy to alleviate severe (pan-) cytopenias [15, 39–41].

The extensive validation of the HT score demonstrates the importance of pre-CAR-T hematopoietic reserve and baseline inflammatory state for the subsequent development of toxicity and early progression in patients receiving BCMA-directed CAR-T. Systemic inflammatory markers have been linked to tumor interferon signaling and suppressive myeloid cells, which can blunt the CAR T-cell expansion necessary for efficient eradication of tumor cells [42, 43]. However, it remains to be studied if similar resistance mechanisms extend to myeloma patients [44]. The poor prognostic impact of cytopenia may be of two-fold origin. On the one hand, cytopenia likely reflects poor disease biology and poor marrow reserve due to multiple prior treatments (including alkylators) or plasma cell infiltration of the bone marrow (Table 1) [45–47]. On the other hand, long-lasting cytopenias may prevent myeloma patients from receiving efficacious post-relapse therapy, such as novel bispecific antibodies or allogeneic CAR-T products, as incomplete count recovery represents a common study exclusion criterion [48–53].

Key limitations of this study include the retrospective nature and limited follow-up. While the inclusion of multiple sites across different health care settings and countries represents a strength of the analysis, this likely resulted in heterogeneity in terms of toxicity management strategies. A further limitation was that response assessment was not performed centrally (by independent review committee). Despite these limitations, we see several salient clinical implementations of the CAR-HEMATOTOX score. The score can be easily calculated using the online calculator and enables early risk-stratification of severe toxicity and poor treatment response *prior* to lymphodepletion. As a result, future studies may explore HT-adapted strategies for anti-infective prophylaxis and early G-CSF use, so as to mitigate the risk of severe infections [17, 37]. Considering their overall low risk of severe toxicity, HT^low^ patients represent an attractive population to explore antibiotic-sparing measures that could prevent deleterious effects on the gut microbiome [54]. Integrating longitudinal assessments of serum procalcitonin may help to identify particularly low-risk patients in the context of CRS (e.g. HT^low^ patients with non-elevated serum procalcitonin at time of first fever) [22, 55]. Low-risk candidates may also be considered for outpatient CAR T-cell application [56, 57]. On the other hand, patients with a high HT score likely benefit from intensified monitoring, anti-infective prophylaxis, early G-CSF, and awareness for a potential stem cell boost. Considering the expected approval of several T-cell engaging therapies for clinical use in r/r multiple myeloma [48, 50–52], future studies may also evaluate the utility of the HT score for this treatment modality.

## Conclusions

In conclusion, the CAR-HEMATOTOX score represents a potent risk-stratifier for severe toxicity and clinical outcomes prior to lymphodepletion, warranting further prospective validation. The score could enable tailored interventions for CAR-T-related toxicity according to the individual risk profile of each patient, and help identify CAR-T candidates in need of combinatorial and/or novel therapeutic strategies.

## Supplementary Information

**Additional file 1.** Supplementary Methods, Tables and Figures.

## Data Availability

For original data and material, please contact kai.rejeski@med.uni-muenchen.de.
