# Supplementary material for: The CAR-HEMATOTOX score as a prognostic model of toxicity and response in patients receiving BCMA-directed CAR-T for relapsed/refractory multiple myeloma
Source: J Hematol Oncol. 2023 Jul 31;16:88. doi: 10.1186/s13045-023-01465-x (PMC10391746; doi:10.1186/s13045-023-01465-x)
Supplement: Supplementary file 1 — Additional file 1. Supplementary Methods, Tables and Figures. [file 13045_2023_1465_MOESM1_ESM.pdf]

Supplementary Material

Supplementary Methods

Patients and data collection

The following CAR-T centers participated across three countries (USA, Germany, France): LMU Munich (Munich, Germany), TU Munich (Munich, Germany), Hospices Civils de Lyon (Lyon, France), Mayo Clinic Rochester (Minnesota, USA), Mayo Clinic Jacksonville (Florida, USA), Moffitt Cancer Center (Tampa, Florida). A uniform data collection form with embedded data dictionary was provided to all participating centers in May 2022 by the coordinating center (LMU Munich), along with an example on guidelines for data collection. All sites returned data to the coordinating center. A quality control check was performed by the coordinating center and queries were issued for missing data or data that did not follow the format specified in the data collection form. All retrospective data were collected with institutional review board approval: LMU Munich: Project Nr. 19-817; Lyon: CNIL, approval No. 18-076, Mayo: 21-006535, Moffitt Cancer Center: Advarra Pro00046602.

Definitions of the neutrophil recovery phenotypes

1) Quick: sustained neutrophil recovery without a second dip below an ANC <1000/µL.
2) Intermittent: neutrophil recovery (ANC>1500/µl) followed by a second dip with an ANC <1000/µL after day 21.
3) Aplastic: continuous severe neutropenia (ANC<500/µL) ≥14 days.

Supplementary Tables

Prophylaxis	Moffitt Cancer Center	Mayo Clinic	LMU University Hospital
**Antiviral prophylaxis**			
Medication	Acyclovir 800mg PO BID	Acyclovir 400mg PO BID	Acyclovir 400mg PO BID
Started	Day -1 or when ANC < 500/uL (whichever occurs first)	Day 1	Day -5
Stopped	Day 360 (12 months)	When CD4 > 200	When CD4 > 200
**Antibacterial prophylaxis**			
Medication	Levofloxacin 500mg PO daily	Levofloxacin 500mg PO daily	None
Started	When ANC < 500/uL	When ANC < 500/uL	None
Stopped	When ANC > 500/uL	When ANC > 500/uL for 2 consecutive days	None
**Antifungal prophylaxis**			
Medication	Fluconazole 400mg PO daily	Fluconazole 400mg PO daily	Only in high-risk patients (s. below)
Started	When ANC < 500/uL	Day 1	Only in high-risk patients (s. below)
Stopped	When ANC > 500/uL	Day 30	Only in high-risk patients (s. below)
Other considerations	Voriconazole 200mg PO BID for history of invasive fungal infection, neutropenia > 14 days on admission, anticipated steroid duration > 14 days, or patients with ANC < 500/uL for > 14 days post-CAR T-cell infusion with long-term cytopenias anticipated		Posaconazole 300mg PO daily once ANC < 500/uL and continued until stable count recover in case of baseline cytopenia, history of invasive fungal infection, or anticipated steroid duration > 3 days
**PCP prophylaxis**			
Medication	Bactrim SS 1 tablet PO daily or alternative**	Bactrim SS 1 tablet PO daily (for patients whom Bactrim is contraindicated, pentamidine if preferred. If pentamidine is contraindicated, then atovaquone)	Bactrim DS 1 tablet PO three times weekly or alternative**
Started	Day 30	Day 1	Day -5
Stopped	Day 180 (6 months) or when CD4 count > 200/uL	when CD4 count > 200/uL	when CD4 count > 200/uL

Table S1. Details of antibiotic prophylaxis
PO: By mouth. BID: Twice daily. ANC: Absolute neutrophil count. IV: Intravenous. PCP: Pneumocystis pneumonia (*Pneumocystis jirovecii*). SS: Single strength tablet (400mg/80mg). DS: Double strength tablet (800mg/160mg).
*Alternatives include isavuconazole 372mg PO daily, micafungin 100mg IV daily over voriconazole due to potential for neurotoxicity.
**Consider atovaquone 1500mg PO daily if patient positive for toxoplasma IgG. If patient negative for toxoplasma IgG consider atovaquone 1500mg PO daily, dapsone 100mg PO daily or pentamidine 300mg inhaled or IV every 28 days.

Table S1: Continued

Prophylaxis	CHU LYON SUD	TUM
**Antiviral prophylaxis**		
Medication	Valaciclovir, PO, 500 mg BID	Valacyclovir 500mg: 1-0-1
Started	Prior to CAR T-cell infusion	At least ANC < 500/µL, or dependent on long-term treatment with Imids, PI and other antineoplastic therapies
Stopped	18 months	At least 6 months after CAR T-cell treatment, in dependence of ongoing B/T-cell defects
**Antibacterial prophylaxis**		
Medication	No standard prophylaxis	No standard prophylaxis
Started		
Stopped		
**Antifungal prophylaxis**		
Medication	No standard prophylaxis	No standard prophylaxis
Started		
Stopped		
Other considerations		
**PCP prophylaxis**		
Medication	Bactrim DS 1 tablet PO three times weekly	Bactrim DS 1 tablet PO three times weekly
Started	Before CART cells	At least ANC < 500/µL, or dependent on long-term treatment with Imids, PI and other antineoplastic therapies
Stopped	Month 18	At least 6 months after CAR T-cell treatment, in dependence of ongoing B/T-cell defects

Characteristic	All Patients (n=113)	CAR-HEMATOTOX Score		*p*
		Low (n=63)	High (n=50)	
**CRS maximum grade – n (%)**				0.14
0	18 (16%)	11 (17%)	7 (14%)	
1 – 2	89 (79%)	51 (81%)	38 (76%)	
3 – 5	6 (5%)	1 (2%)	5 (10%)	
**ICANS maximum grade – n (%)**				**<0.001**
0	90 (80%)	57 (91%)	33 (66%)	
1 – 2	15 (13%)	6 (9%)	9 (18%)	
3 – 4	8 (7%)	0 (0%)	8 (16%)	
**Treatment for toxicity – n (%)**				
Antibacterial prophylaxis use (e.g. fluoroquinolone, ciprofloxacin or levofloxacin)	90 (80%)	48 (76%)	42 (84%)	0.35
Steroid use	44 (39%)	18 (29%)	26 (52%)	**0.01**
Tocilizumab use	87 (77%)	48 (76%)	39 (78%)	> 0.9
Anakinra use	2 (1.8%)	0 (0%)	2 (4%)	0.19
ICU admission	6 (5.3%)	1 (1.6%)	5 (10%)	0.086

Table S2 Coincident immunotoxicity and management
Severity of CRS and ICANS and distribution of toxicity management by CAR-HEMATOTOX score. P-values determined using Fisher's exact tests for categorical variables.

Response Criteria	All Patients (n=108)*	CAR-HEMATOTOX Score		*p*
		Low (n=60)	High (n=48)	
**Best Response by Day 90 according to IMWG criteria – n (%)**				0.10
Complete Response (CR) or stringent CR (sCR)	43 (40%)	17 (45%)	16 (33%)	
Very Good Partial Response (VGPR)	20 (19%)	15 (25%)	5 (10%)	
Partial Response (PR)	25 (23%)	11 (19%)	14 (29%)	
Stable Disease (SD)	12 (11%)	5 (8%)	7 (15%)	
Progressive Disease (PD)	8 (7%)	2 (3%)	6 (13%)	
**Response Rates**				
Objective Response Rate (≥PR)	88 (81.5%)	53 (88.3%)	35 (72.9%)	**0.048**
≥ VGPR	63 (58.3%)	42 (70.0%)	21 (43.8%)	**0.01**
≥ CR	43 (39.8%)	27 (45%)	16 (33.3%)	0.24

Table S3 Response to BCMA CAR-T according to CAR-HEMATOTOX score
Comparison of all response groups was performed with Chi-squared test, while direct comparison of response rates was analyzed using Fisher's exact test.
* 5 patients did not have response assessment data or assessment was not evaluable for response.

**Characteristic**	**n**	**Univariate**		**Multivariable**	
		**HR (95% CI)**	***p***	**Adjusted HR (95% CI)**	***p***
Estimated GFR <60 ml/min prior to lymphodepleting chemotherapy	31/113	1.8 (1.01-3.3)	**0.046**	1.4 (0.8-2.7)	0.27
LDH greater than upper limit of normal	35/113	2.0 (1.1-3.4)	**0.02**	1.1 (0.6-2.0)	0.79
ECOG performance status ≥2	12/113	4.1 (2.0-8.4)	**<0.001**	2.0 (0.9-4.4)	0.08
Plasma cell infiltration of the bone marrow ≥50%	28/113	2.4 (1.3-4.3)	**0.004**	1.6 (0.9-3.0)	0.15
CAR-HEMATOTOX score ≥2	50/113	4.7 (2.5-8.7)	**<0.001**	3.5 (1.7-6.9)	**<0.001**

Table S4 Univariate and multivariable Cox Proportional Hazards Model for PFS
The number of patients within each risk group is depicted together with the hazard ratio (HR) and 95% confidence interval (CI) and p-value.

**Characteristic**	**n**	**Univariate**		**Multivariable**	
		**HR (95% CI)**	***p***	**Adjusted HR (95% CI)**	***p***
Estimated GFR <60 ml/min prior to lymphodepleting chemotherapy	31/113	1.9 (0.8-4.4)	0.1	1.1 (0.4-2.8)	0.81
LDH greater than upper limit of normal	35/113	2.1 (0.9-4.7)	0.086	1.03 (0.4-2.4)	>0.9
ECOG performance status ≥2	12/113	7.5 (3.0-18.9)	**<0.001**	3.8 (1.4-10.2)	**0.008**
Plasma cell infiltration of the bone marrow ≥50%	28/113	3.1 (1.4-7.2)	**0.007**	1.9 (0.8-4.6)	0.18
CAR-HEMATOTOX score ≥2	50/113	6.2 (2.3-16.9)	**<0.001**	3.5 (1.1-11.2)	**0.03**

Table S5 Univariate and multivariable Cox Proportional Hazards Model for OS
The number of patients within each risk group is depicted together with the hazard ratio (HR) and 95% confidence interval (CI) and p-value.

Supplementary Figures

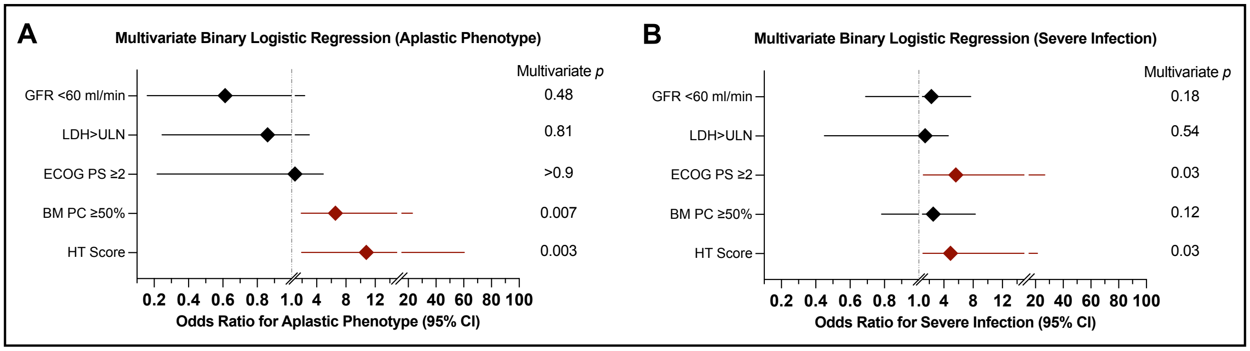

Figure S1. Multivariate binary logistic regression analysis of baseline patient features for the aplastic phenotype and severe infections
All covariates were assessed prior to lymphodepleting chemotherapy. The adjusted multivariable p-value is provided.

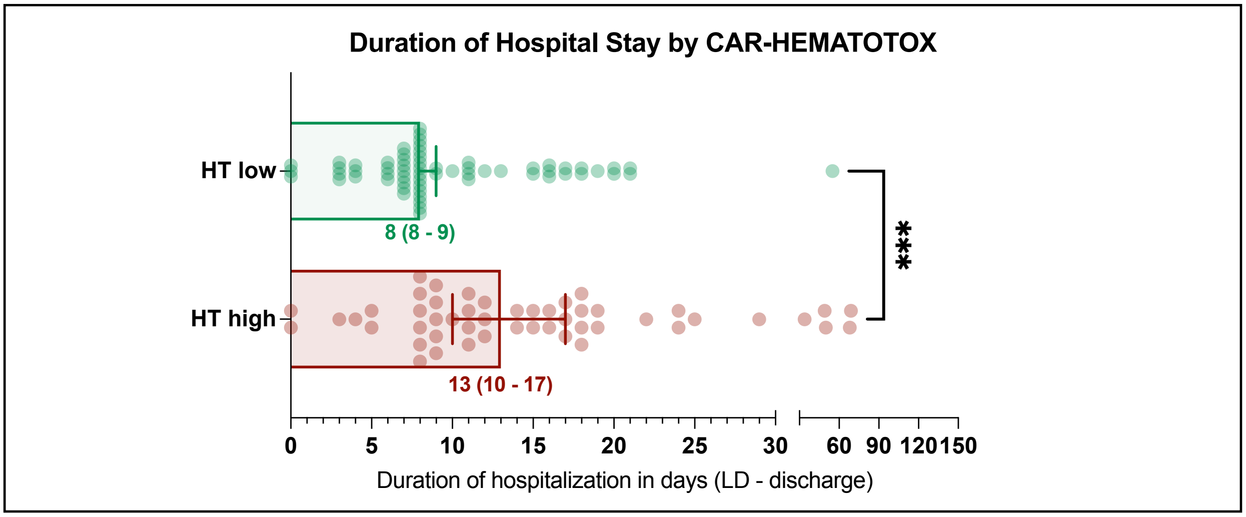

Figure S2. High CAR-HEMATOTOX scores were associated with prolonged hospital stay
Length of hospitalization was determined from start of lymphodepletion until discharge from first hospital admission.

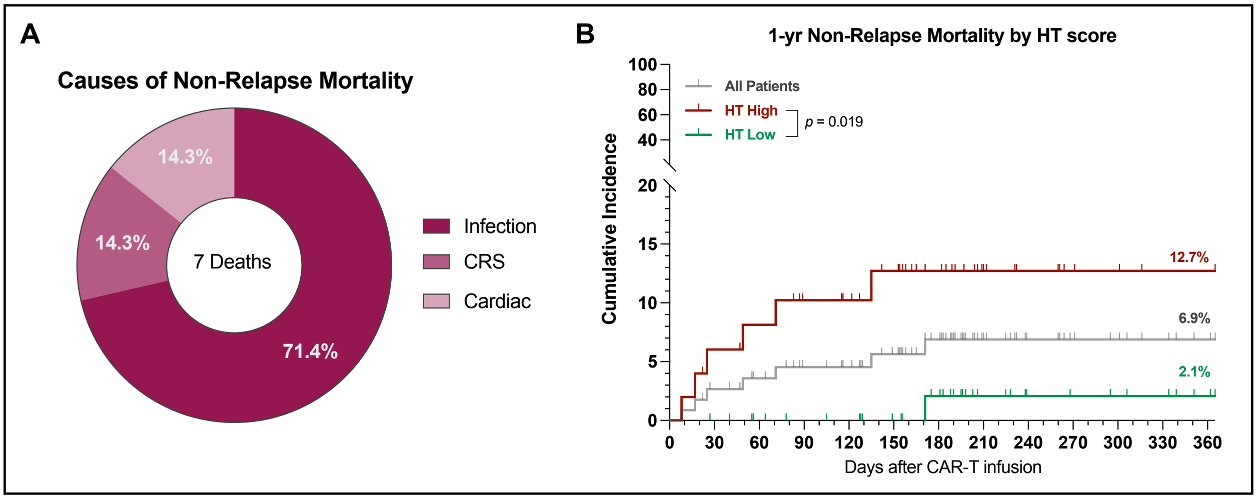

Figure S3. The CAR-HEMATOTOX identifies patients at risk for higher non-relapse mortality after BCMA-directed CAR-T for R/R multiple myeloma

A Overview of the contributing causes of non-relapse mortality (NRM). Seven deaths were attributed to a non-relapse associated for the entire study follow-up. **B** On-year NRM across all patients (gray) and in patients with a HT score ≥2 (high, red) vs. 0-1 (low, green) patients. The p-value of the Mantel-Cox log rank test comparing HT risk groups is depicted.

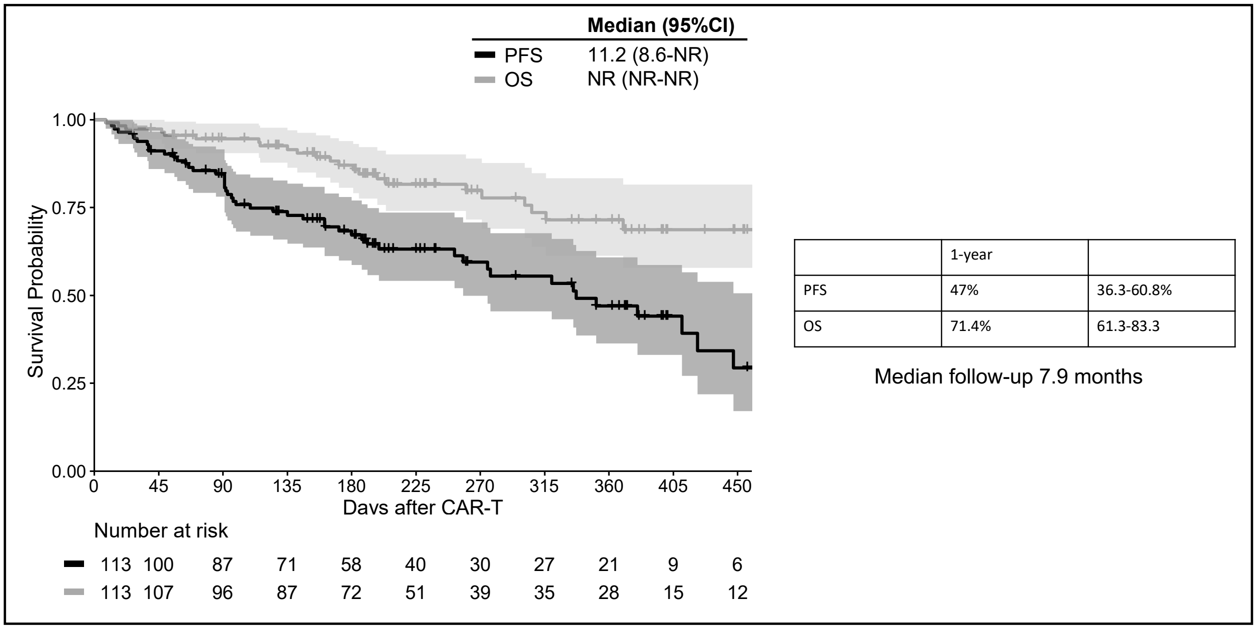

	1-year	
PFS	47%	36.3-60.8%
OS	71.4%	61.3-83.3

Figure S4. Survival outcomes in a real-world cohort of r/r multiple myeloma patients treated with BCMA-directed CAR T-cell therapy

Kaplan-Meier estimates for progression-free survival (PFS, dark grey) and overall survival (OS, light grey). The table indicates the 1-year PFS and OS rate with 95% confidence interval. The median follow-up time was determined using the reverse Kaplan-Meier method.
